# The role of embedded public health researchers in local government: Identifying markers of changes in relationships and how evidence is applied in policy and practice

**DOI:** 10.12688/wellcomeopenres.25838.1

**Published:** 2026-02-20

**Authors:** Lisa Dowling, Amy Barnes, Rob Southall-Edwards, Rachel Williams, Hibah Iqbal

**Affiliations:** 1 Bradford Institute for Health Research, Bradford, England, UK; 2 Public Health and Society, Health Sciences, University of York, York, England, UK; 3 Institute of Health and Well-being, University of Suffolk, Ipswich, England, UK; 4 School of Sport Rehabilitation and Exercise Sciences, University of Essex, Colchester, England, UK; 5 South West Yorkshire Partnership NHS Foundation Trust, Wakefield, England, UK; 6 Essex County Council, Chelmsford, England, UK

**Keywords:** Embedded research; Public health; Local government; Evidence-informed policy

## Abstract

**Background:**

Local government is central to addressing determinants of public health and must use evidence to make effective decisions. In England, local government typically does not have research infrastructure to enable this; an emerging strategy to overcome this challenge is embedding public health researchers. Expectations of embedded researchers (ERs) are often ambitious and long-term (i.e. changing culture towards evidence), with markers of change lacking, making it difficult to measure progress. This study aimed to: (i) better understand the rationale for ERs, and (ii) put forward a framework to track changes resulting from ER roles.

**Methods:**

A qualitative, self-reflective study was carried out by the authors as five ERs. Data collection took place between April 2023-January 2024 in three stages: (1) initial discussion and written reflections, (2) reflective memo-writing, and (3) peer-to-peer interviews. Data was analysed using a hybrid approach, thematic analysis and the Framework Method, alongside an iterative process of interpretation through team discussion.

**Results:**

A framework about how ERs can contribute towards changes in evidence application in local government was developed. Relationships were identified as key. Four common relational strategies by ERs were identified to enable change: (1) reflect on context and tailor communication approaches, (2) collaborate across boundaries, (3) be visible/accessible and (4) create space to reflect and learn. Short-term markers of changes in relationships (e.g. ER expertise is seen, valued, and trusted, and relational networks are built) and markers of subsequent changes in evidence use (e.g. mindsets that seek research and evidence, critical thinking) were identified.

**Conclusions:**

A novel ER framework was developed (four relational strategies with markers of subsequent change), which can be used and tested in future research, evaluation, and practice: to develop clearer theories of change for ER initiatives, help ERs develop confidence in their roles and/or develop clearer ER job specifications and objectives.

## Plain Language summary

Local governments play an important role in improving public health, but in England, they often lack the research resources needed to make evidence-based decisions. One way to address this challenge is by placing public health researchers directly within local government teams. These embedded researchers (ERs) are often expected to create long-term changes, like encouraging a culture that uses research to inform decisions. However, it can be hard to measure progress in the short-term. This study aimed to (1) examine the purpose of ERs in local government and (2) develop a framework to track short-term changes resulting from ER work.

The study was carried out by five ERs between April 2023 and January 2024. Data was collected through discussions, personal reflections, written memos, and interviews among the researchers and analysed using a structured approach to identify common themes.

A framework about how ERs contribute towards long-term changes in evidence use in local government was developed with relationships identified as key. Four common approaches were found to enable long-term change: (1) adapting communication to the local context, (2) collaborating across different teams, (3) being visible and accessible to staff and (4) creating opportunities for reflection and learning. In the short-term, relationship changes can be identified by markers such as ER expertise being seen, valued, and trusted, and ERs helping to build networks within the organisation. Subsequent change in evidence use can be identified by markers such as staff starting to seek out research and evidence, think critically and use evidence more in decision-making.

The study produced a new framework showing how ERs can support evidence use in local government. It outlines practical strategies and early markers of change. This framework can help future ERs understand their role, guide their work, and be tested in research and practice to improve evidence-informed decision-making.

## Introduction

Local government plays a pivotal role in protecting and improving population health and wellbeing through public health strategies and action on wider determinants of health. ^1, 2^ Creating, synthesising and applying research evidence is central to effective decision-making, yet, in England, local government typically does not have access to the necessary research infrastructure to enable this. ^3^ Evidence-to-practice research systems exist in England, but these predominantly exist outside of local government in healthcare, community, and academic partnerships. ^3^ Whilst research in these outside environments aims towards translation by affecting policy-making or frontline practice, ^4^ the translation of evidence into policy and practice is complex. ^5, 6^ Research can be hard to locate and understand, ^6^ may not carry actionable messages, ^7^ ‘fit’ the local context ^4^ or be available when decisions are required. ^6^ These issues are further compounded by the complexity of integrating and synthesising different types of evidence with contextual factors such as political preferences, community pressures, and emergent local issues, and into decisions which need to be made through well-governed democratic processes. ^8– 10^

One strategy proposed to overcome these challenges is the development of partnerships between local government and academic organisations, to enable knowledge production within the realities of policy and practice. ^4, 11^ Six different types of partnership structure have been described which can co-exist and evolve over time, or which can be research- or activity-specific, ^3^ with embedded research promoted as a promising initiative within these structures. Although frameworks and protocols have been developed to enable embedded research, a recent review highlighted a high degree of complexity and nuance within and between these initiatives across disciplines and settings, with over 108 definitions reported, suggesting a continuum of approaches. ^12, 13^ Embedded researchers - in which one or more researchers are involved in generating and supporting the application of research evidence - is the most common approach, albeit with varying levels of ‘embeddedness’. ^12, 14^

Embedded researchers are more common in healthcare settings than in public health and local government. A recent systematic review identified over 40% of reports in healthcare settings compared with only 15% in public health. ^12^ However, the increasing popularity of ‘whole systems’ approaches in response to the complex nature of public health challenges has increasingly led to researchers being embedded at a local government level working with partners. ^15^ Recent investment by the National Institute for Health Research in Health Determinants Research Collaborations in the UK to enable the strengthening of research infrastructure in local government has also led to an increase in the number of embedded public health researcher roles in this policy setting. ^16^

Evidence suggests embedded research can facilitate knowledge co-production with the underlying assumption that when evidence is co-created with stakeholders it will be more relevant to the local context and therefore more easily mobilised into changes in policy and practice. ^11^ In addition to research co-production, embedded researchers are conceived as knowledge brokers who can facilitate and enable research and knowledge exchange through linkage and network creation. ^4, 12^ Others have reflected on factors that enable embedded researchers to achieve success, identifying various process, relational and contextual factors of relevance. Process factors include having a period of engagement prior to embedding and secure funding to allow flexibility in length and depth of embedding, whereas contextual factors such as staff buy-in and motivation are noted as important for success. ^4^ Literature also acknowledges the need for embedded researchers to not only have technical or topical expertise, but also strong social and interpersonal skills such as receptiveness, enthusiasm and communication skills (relational factors). ^4, 5^

When described, markers of change or ‘success’ for embedded research are often ambitious and long-term, referring to culture change, increasing capacity for evidence-informed decision-making, and greater research infrastructure. ^11^ Short-term markers of success are lacking, thus making it difficult for embedded researchers themselves and their host organisations to measure progress towards these longer-term goals. At the time of writing this paper, only one study by Edwards et al. ^17^ identified three short-term outcomes of importance in moving towards organisational culture change in local government: increased awareness, interest, and involvement in research. However, this study was conducted ‘on’ early-stage embedded researchers, only looked for short-term markers relating to the pre-specified outcome of organisational change and was analysed from an ‘outsider’ perspective.

In this paper, we build on the work of Edwards et al. ^17^ by drawing on our own ‘insider’ experiences of working as embedded public health researchers, with a spectrum of embeddedness and experience (one to four years) in two different local government settings. We aim to (i) describe what we, as embedded researchers, identify as the common rationale for our work despite the heterogeneity of our roles, and (ii) put forward a novel framework that we suggest can be used to track short-term changes resulting from embedded researcher roles, thus enabling a better understanding of progress towards longer-term outcomes of evidence-informed decision-making.

## Methods

### Embedded researchers (Participants)

The paper draws on our experiences as five embedded public health researchers who are currently or have recently (within the last 18 months) been embedded within local government settings ( Table 1). In terms of similarities, we have all trained in academic research methods, hold degrees, worked in a hybrid way in our host setting and were allocated specific time to engage as an embedded researcher. Three of us were Senior Research Fellows and two of us were Evaluation Researchers. Our roles involved supporting research and/or evaluation through advising, developing, and delivering research/evaluation, facilitating knowledge exchange, developing partnerships, and fostering collaboration. However, we worked in different types of local government settings (e.g. metropolitan district council vs. county council), had different levels of ‘embeddedness’ as a researcher (see Table 1 and Findings section below), and had been in post for different amounts of time (one to four years). In sharing this information, we have been mindful of protecting our anonymity in relation to quotes used below and the integrity of our programmes of work, whilst providing relevant contextual information for readers to understand the experiences that have informed the findings shared in this paper.

**Table 1.** Embedded research roles and experience.

Researcher	Embedded role
Res1	**Local government setting:** Metropolitan District Council **Dates:** October 2022 - present **Role Type:** Senior researcher; part-time; hybrid working (max one day per week on local government site) **Embedded Role:** Based at University with regular (daily remote; weekly face-to-face) working with local authority staff **Purpose:** Developing partnerships between universities and local authority to ensure research informs policy-practice. Advising, developing, and delivering research-evaluation, acting as knowledge broker, promoting research-evaluation.
Res2	**Local government setting:** County Council **Dates:** March 2022 – Present **Role Type:** Member of evaluation team; full-time; hybrid (max one day per week on local government site) **Embedded Role:** Based within local authority, conducting realist evaluation **Purpose:** Focusing on collating and building evidence and process learning reports using realist evaluation. Supporting building evaluation capacity within the wider team. Building relationships to influence evaluation culture change.
Res3	**Local government setting:** Metropolitan District Council **Date:** October 2022 - Present **Role Type:** Senior Researcher; full-time; hybrid (max one day per week on local government site) **Embedded Role:** Joint academic-local authority role with managers at both sites. **Purpose:** Supporting research, evaluation: advising, developing, and delivering evaluations, acting as knowledge broker, promoting research. Co-producing evaluations, shifting thinking towards evaluation perspective; enabling collaboration.
Res4	**Local government setting:** County Council **Date:** March 2022 – March 2023 **Role Type:** Member of evaluation team; full-time; hybrid (max one day per week on local government site) **Embedded Role:** Based within local government conducting realist evaluation **Purpose:** Increase research and evaluation capacity, assist with knowledge exchange to enable evidence-based decision-making in delivery.
Res5	**Local government setting:** Metropolitan District Council **Date:** August 2020 - April 2024 **Role Type:** Senior Researcher; full-time; fixed-term (4 years); hybrid (one day per week on local government site) **Embedded Role:** Fully embedded, line management from Deputy Director of Public Health. **Purpose:** Leading the evaluation of a local authority programme to help inform its development and delivery. Advising and supporting research and evaluation activities; building research capacity; fostering collaboration between academia and practice.

### Data collection

A favourable ethical opinion to conduct this research was provided by the University of Suffolk Research Ethics Committee (RETH(S)23/011). Researchers provided informed written consent prior to participating in the research. Data collection took place between April 2023 and January 2024, and consisted of three main stages: (1) initial discussions and written reflections, (2) writing reflective memos, and (3) peer-to-peer interviews.

***Initial discussions and written reflections***

We organised informal discussions (online via Microsoft Teams) about our embedded research roles and subsequent barriers, enablers, and achievements we experienced. We completed a process of idea mapping using an online whiteboard (Jamboard) to identify areas for further exploration. These were: an overview of the embedded researcher role; reasons for bringing in an embedded researcher (i.e. expectations and theories about what an embedded researcher brings, can or could achieve); and how we measure and perceive the success of our roles. We completed personal and written reflections on these areas individually. Written reflections were discussed by all of us together in a series of online meetings (via Microsoft Teams). Using an iterative process, common functions of our embedded researcher role were identified (relationships, capacity, and culture change) to provide a focus for more in-depth reflective memos.

***Reflective memos***

We completed in-depth, evidence-based memos ( **Extended Data**) exploring the topics of relationships, capacity and culture change, including examples of things we were working on, what this had led to and examples of perceived ‘successes’ or issues we experienced. Memos were reviewed by all of us and comments or questions noted where we felt further detail could be added. As a group, we decided that additional data collection was warranted to provide more in-depth insight about our roles and so we organised peer-to-peer interviews to enable this.

***Peer-to-peer interviews***

Semi-structured one-to-one peer-to-peer interviews (online via Microsoft Teams) were used to further clarify and understand our embedded roles. Reviewing our structured reflections informed the development of a semi-structured interview guide (see **Extended Data**) and comments or questions noted on each individual researcher’s structured reflections (as above) were used to guide further questioning during the interviews. To avoid assumptions being made due to prior knowledge, we organised the interviews so that we were paired with someone outside of our local government setting. Interviews were recorded (using Microsoft Teams) and initial transcripts generated using the platform's built-in transcription feature. We reviewed and cleaned our respective transcripts to ensure accuracy, correcting any errors or inconsistencies. Cleaned transcripts were used for analysis.

### Data analysis

We analysed our reflective memos and peer interviews using a hybrid approach, combining Braun and Clarke’s (2006) thematic analysis with the Framework Method (Gale et al., 2013). This process involved the following:

1) *Familiarisation with the data*: We read and reread all the reflective memos and interview transcripts to gain a comprehensive understanding of the data.
2) *Generating initial codes:* Our coding process was iterative with two researchers (Res3 & Res5) working collaboratively to generate labels (codes) to capture key concepts, themes and ideas (e.g. expectations about ER roles, barriers, proving yourself and judging successes) *.* The collaborative approach allowed for immediate discussion and resolution of any discrepancies in interpretation, enhancing the consistency and depth of the initial coding process.
3) *Developing an analytical framework:* After initial coding, three researchers (Res1, Res3 & Res5) met to discuss and agree upon a set of final codes. Codes were then grouped into high-level themes (e.g. ER roles, strategies to bring about change, markers of success) to form a preliminary analytical framework. We met as a whole team during this process to discuss the codes and themes, and to agree upon the final analytical framework.
4) *Applying the analytical framework:* The analytical framework was applied to all transcripts, using the existing categories and codes. New codes that emerged were added to the framework.
5) *Charting data into the framework matrix:* Data were summarised by theme and charted into a matrix (using Microsoft Excel), with each column representing a case (i.e., an individual researcher’s reflections) and each row representing a code or category. Researchers worked in pairs to chart data and themes, reviewing and discussing each other’s work to ensure consistency in interpretation and maintain data integrity.
6) *Interpreting the data to develop meaning:* We engaged in an iterative process of team discussion and reflection to refine our understanding of each high-level theme. To ensure the rigour of our analysis, we regularly had debriefing sessions to provide challenge, ensure consistency in our interpretation and reach consensus on findings. Through this process, we, for example, developed a better understanding of markers of change and were able to identify five key markers and identify and assign examples of change to each of these. We maintained an audit trail of our analytical decisions and engaged in reflective discussions, critically examining how our experiences might influence our interpretations.

## Findings

### Introducing our ER roles

Our roles all involved working in embedded ways with local government partners: we all identified as researchers who were ‘placed’ (i.e. located in some way) in this setting and were regularly supporting staff and/or carrying out research within it. Beyond this, however, the nature and extent of our ‘embeddedness’ differed in relation to: (1) our ‘insider-outsider’ status, i.e., whether we primarily worked ‘inside’ the local authority as a paid (n=2; Res2, Res4) or honorary (n=2; Res3, Res5) member of staff, or further ‘outside’ as a close partner (n=1; Res1) (with discussions around honorary status); (2) our affiliation with an academic institution, i.e., whether employed by an academic institution (e.g. University or NHS research institute) (n=3; Res1, Res3, Res5) or employed by a local government authority but supported by an external academic consortium and recognised as having the ‘academic/research competency’ (n=2; Res2, Res4).

Despite our different types of ‘embeddedness’, it became clear that we tended to carry out similar research- or evidence- (knowledge) related tasks. For example, our work involved: advising and/or supporting local authority staff on how to analyse information (e.g. poverty-proofing school audits, creating case studies to demonstrate impact of a cycling scheme); collating different forms of evidence (e.g. locally-generated evidence on healthy urban places, best practice for whole-school approaches, and ultra-processed foods); supporting evidence use (e.g. evaluative evidence) in policy development or programme design (e.g. bringing evidence to strategic working groups) or directly supporting the evaluation of funded programmes (e.g. food strategy, school streets, children’s holiday healthy lifestyle programmes).

There were similarities in relation to the perceived rationales for our roles and our expectations for change. We were all broadly able to describe the overall, high-level purposes of what we were recruited to do: build relationships, capacity, and culture for carrying out research and using evidence in policy and practice. However, a common experience was some lack of clarity about what these concepts meant to us or in the local government environments that we were working in, how we were expected to achieve these outcomes as individuals, and therefore the expectations for what we would be working on, our deliverables and contribution to wider change. In our reflections we commented on the lack of clarity and unbounded nature of our roles. As one of us explained:

*“It is difficult to know exactly what changes people wanted to see - the role was new and there were no clear objectives for me to meet.” [Res3, memo]*

The lack of clarity led to us questioning our role and contribution, as well as how this was perceived by those around us in the local government setting. The ambiguity made it challenging to evaluate our own contributions. As two of us commented:

*“I find sometimes it’s hard to, kind of, think ‘well, what is my contribution there?’” [Res1, interview]*

“ *I didn't really know what I was letting myself in to. I didn't think I would be needing to prove myself…” [RES2, interview]*

### Relational and research-evidence changes that embedded research(ers) can help bring about

Through the reflective process and ‘safe space’ we created within this research, we developed greater clarity about our embedded roles: not only the strategies we used day-to-day, but also identifying with greater specificity the outcomes that we sought to achieve in our roles.

**Figure 1.**
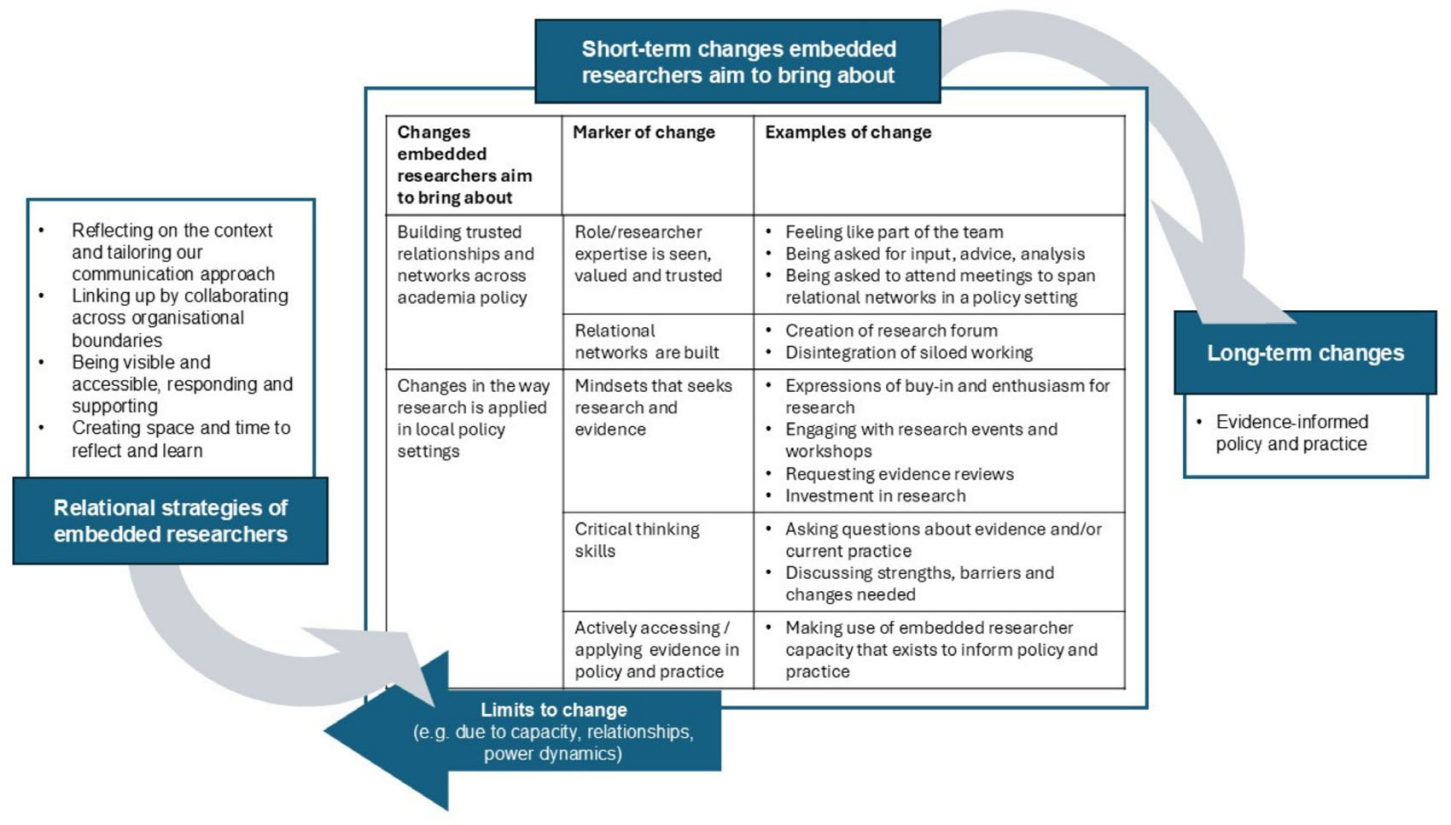
Visual diagram of the relational strategies used by embedded researchers and research-evidence changes that embedded research(ers) can help bring about.

Although at the start of our reflective journey, we initially felt that our roles broadly aimed to build relationships, capacity, and culture, through memo-writing and interviews we gained greater specificity around the purpose of our roles. It became clear that building relationships was a key pathway to sharing academic evidence (e.g. rapid evidence reviews), sharing or developing methodological knowledge (e.g. ripple effect mapping), and promoting critical thinking about existing practices, so as to generate new knowledge about how to apply evidence locally (e.g. asking questions about how communities are or are not engaged currently and therefore how the knowledge could be better used in local strategy development) (see Figure 1). We identified four common relational strategies that we had all used to try and achieve these changes.

1. ***Reflecting on the context and tailoring our communication approach***

We all spent time and emotional labour in our roles reflecting on the different staff teams and policy settings we were working in; for example, being aware of the extent or quality of local government’s existing relationships and past experiences of working with researchers. From this, we tailored our approach and how we communicated, adapting the language we used in council or policy-related community meetings, emails or reports to be more or less academic, or using commonly understood terminology, as appropriate to the Council department or public health topic. We did this to demonstrate respect for people's existing knowledge, status, and authority, but also carefully used language to try and ‘fit in’, whilst generating interest and enthusiasm in evidence or methods, and confidence in our potential contribution. In some cases, this also involved consideration of what we wore, particularly if working in community settings. As one of us explained:

*“I went and did some data collection […] We sort of dressed down, no lanyards [*… *] got rid of the clipboards. And people spoke to us, whereas before I'd tried to go along with, like, a clipboard and looked more official and I just got, like, what do you want?” [Res4, interview]*

2. ***Linking up by collaborating across organisational boundaries***

The second strategy involved connecting local government staff with other researchers to enable access to expertise and knowledge. Practically, this involved inviting or connecting other researchers or policy stakeholders into meetings (e.g. to develop theories of change and discuss evaluation approaches), joining ‘external’ working groups (e.g. anti-poverty groups), and connecting partners via email to set up new relationships; therefore, creating routes to access knowledge and expertise. The intention here was that helping to build these wider relational networks would enable access to evidence or research skills that could support evidence into practice. As one of us explained:

*“so, if I have a fortuitous conversation with someone in the Council, I would try and do some linking […] weaving of other relationships, through being, like, embedded and having those connections.” [Res1, interview]*

3. ***Being visible and accessible, responding and supporting***

Our third strategy involved being present: consciously spending time with staff, communicating in ways that showed we were accessible and could be contacted easily to provide input or advice, *“to be visible, is to be valued” [Res4, interview].* We focused on visibility and accessibility so that we would be perceived as someone who understands the local government context and become a trusted route for sharing evidence to inform practice. Both virtual and physical presence and visibility were important:

*“You don't get that same kind of relationship building do you? Like over the video or something*… *you can't build relationships or have the same kind of trust or connection with somebody by working remotely as you can actually just getting together around a table and having a chat.” [Res5, interview]*

We also focused on supporting staff, for example, answering questions, giving advice in the ‘day-to-day’, sending on evidence reviews, developing logic models and outcome markers. As one of us indicated: *“It often ends up that we're not just doing our own work, but we're also turned to for other little bits of research here and there because we are just part of the team, just like any other member.” [Res2, interview]*

4. ***Creating space and time to reflect and learn***

Our final strategy involved creating formal and informal spaces outside staff members’ routines to promote reflection about how and why evidence is or could be used in decisions. Formal spaces included ‘share and learn’ workshops and ripple effect mapping sessions and informal spaces included ad hoc conversations, such as at the end of meetings or while travelling between working environments (“ *conversations that aren't scheduled”* and not *“[a] transaction for a specific purpose” [Res3, interview]).* In both spaces, we focused on asking questions in ways that would support reflection about current practice, learning about different types of evidence or methods.

*“I guess through trial and error of trying various different things and seeing how people engaged with them, we started to […] feedback information […] through workshop activities […] As a way of […] having people actively engage in it more [*… *] to either reinforce what we were already seeing or make it easier to understand how it would be part of their role.” [Res4, interview]*

### Markers of change

With this understanding about how relational strategies were core to our understanding of change, we were able to identify markers and examples ( Table 2) of changes in **relationships** and subsequent changes in **the way that research and other forms of evidence were applied in local government policy and practice**.

**Table 2.** Summary of markers of change with practical examples.

Key changes we, as embedded researchers, aim to bring about	Marker of change	Examples of change we identified
Building trusted relationships and networks across academia policy	Role/researcher expertise is seen, valued and trusted	● Feeling like part of the team ● Being asked for input, advice, analysis ● Being asked to attend meetings to span relational networks in a policy setting
	Relational networks are built	● Creation of research forum ● Disintegration of siloed working
Changes in the way research is applied in local policy settings	Mindsets that seek research and evidence	● Expressions of buy-in and enthusiasm for research ● Engaging with research events and workshops ● Requesting evidence reviews ● Investment in research
	Critical thinking	● Asking questions about evidence and/or current practice ● Discussing strengths, barriers and changes needed
	Actively accessing and applying evidence in policy and practice	● Making use of the embedded researcher capacity that exists in clear examples of evidence-informed policy and practice

### Building trusted relationships and networks across academia and policy

***Role/research expertise is ‘seen’, valued and trusted***

An important marker of relationships changing was feeling like, or having tangible examples of, our role or expertise being recognised, valued or trusted. This marker reflected progress towards building trusted relationships and broadening relational networks across academia and policy. We felt that being valued was an important route to sharing knowledge. In some cases, this marker was reflected in us feeling like we were an integral part of a team: *“Both myself and PH staff express that I’m an integral ‘part of the team’ and the past 3 years have enabled me to build a strong network in [city].” [Res5, interview].* Change was also reflected in tangible examples of ‘being seen’, such as being asked directly by different members of our team or our wider policy network to provide advice, support, and/or analysis, rather than being delegated jobs by a manager. One of us, for example, described how we had been asked by a member of the public health team in the Council to synthesise local research findings to inform the development of a new section of the Local Plan. This led to those findings being incorporated and opportunities for the researcher to provide comments on new sections of text for the Local Plan [Res1, memo]. Another example described how one of us enabled communication with a community partner, bridging the relationship and the way evidence could be shared:

*“I think the relationship was quite strained […] because I wasn't a council employee, but I understood both ways of working, they were then open to that, and that relationship could then happen [*… *]” [Res5, interview]*

***Building relational networks***

Another marker of relationships starting to change was through the establishment of policy-academic collaborations, which drew in knowledge, expertise, and evidence. For example, one of us explained how a forum was organised for partners across academia, policy, and practice to “ *act as a formal and tangible link between research and practice.” [Res5, memo].* Similarly, another one of us explained how we used our networks to bring research capacity into the council by connecting people: *“So not only did we bring in our own capacity, we could tap into the skills and knowledge that were in the wider research team within the council and join things up there” [Res4, memo].*

**Changes in the way that research and other forms of evidence are applied in local authority policy and practice**

***Change in mindset about research:***

One marker of change in the way research and/or other forms of evidence were applied related to a shift in mindset about research. Changes in mindset were identified by us all and were expressed as increased buy-in and enthusiasm amongst people in our network for accessing and using research and evaluation. Practical examples involved staff engaging with research events and workshops, requesting evidence reviews to directly inform policy, and/or talking more about the practical use of research and evaluation in ways that demonstrated it was valued. This included, for example, appreciating and advocating the use of the ripple effects mapping method with stakeholders, or demonstrating a changed openness to learning:

*“we started to [*… *] feedback information from the work we're doing through workshop activities at some of the away days and things [*… *] They were open to the learning and kind of the understanding, the challenges and learning from it […] I felt like I was getting that engagement with some individuals and I would see that as a success of, like, maybe changing culture.” [Res4, interview]*

Another expression of a change in mindset was investing time in research-related activity (e.g. staff members reading and discussing research papers or evidence briefs), as well as actual organisational investments in research effort (e.g. employing more embedded researchers/analysts/intelligence managers, applying for research funding in the local setting).

***Critical thinking skills:***

Critical thinking skills were identified by all researchers as a marker of short-term change in the way research and evidence was applied. This was evidenced in examples of ‘piqued interest’ amongst local government colleagues in research and evaluation findings, colleagues asking more questions about evidence or current practice, and reflecting on strengths, barriers and changes needed - both the positive and the negative:

*“I’ve noticed fellow [*… *] colleagues acknowledging challenges [*… *] things that have gone wrong, questioning how and why, what we can learn. I see this as a success [*… *] because they usually just focus on the positives and I feel a good research culture offers critique, self-awareness in order to be objective and a willingness to learn from things that have not gone well.” [Res2, memo]*

***Actively accessing and applying evidence in policy and practice***

The final marker of change we identified related directly to examples of applying evidence in policy and practice. These were manifestations of evidence-based action and reflected interactions between all the other changes identified above. Examples of applying evidence involved staff members drawing on trusted relationships with us, as well as a mindset that valued research and critical thinking to consider how and whether or not to draw on evidence in a particular setting. Here, one of us described how we had worked with a public health specialist to improve the uptake of a survey about early years development that was lengthy and for which parental completion rates were low (Res3). Through asking probing questions and critical thinking together about the value of the survey and specific questions, it became apparent to the public health specialist that the survey was not fit for purpose and a decision was made to reallocate resources to gather evidence about child development in other ways. Similarly, another of us shared an example of preparing a review for the adult social care team to understand evidence about factors that shaped support for minoritised groups which actively informed a new local strategy (Res1). The process of carrying out the review provided opportunities for the social care team to reflect on how local evidence from communities could best be gathered and discussions informed the way that community engagement and a survey were carried out in practice (e.g. framing of survey questions, how to create effective spaces to engage and with which minoritised groups).

***Limits to change***

Importantly, these examples of applying evidence in policy and practice, and, indeed all the markers of change and strategies of bringing about change, as described above, were influenced by limits to our capacity as embedded researchers, including who and the number of people we were able to interact with as individuals, the capacity of staff and colleagues working in local government settings, as well as historical relationships and power dynamics (e.g. status, hierarchies). In some cases, we experienced issues around censorship or policy partners not applying evidence because there were insufficient resources to make changes, it challenged existing norms and practices and/or associated sensitivities around how applying it would be accepted by others locally.

## Discussion

This paper has presented a novel framework, informed by in-practice reflections, that can be used to track short-term changes (measures of success) that may result from embedded researcher roles. The framework can be used and tested in future research by embedded researchers themselves and their host organisations, to assess its value in better understanding how embedded researchers contribute towards long-term changes towards evidence-informed decision-making.

As indicated in our introduction and the findings section above, there tends to be a lack of clarity surrounding embedded researcher roles in practice, with little guidance on what daily responsibilities or the scope of these roles are or should be, which is an issue highlighted in other research. ^11, 12^ Although we were unclear at the start of this study, during the reflective process we developed greater clarity about our embedded research, as well as the important relational strategies that we had all used to try and bring about change. Despite the heterogeneity of our embedded roles we identified four common strategies, namely: (1) reflecting on the context and tailoring our communication approach, (2) linking up by collaborating across organisational boundaries, (3) being visible and accessible and (4) creating space and time to reflect and learn. These findings reflect wider literature around how embedded researchers produce or facilitate research, broker knowledge ^12^ and respect the local context. ^15^

We suggest that embedded public health researchers can usefully refer to these four relational strategies and the markers of subsequent change identified in our framework to support reflection on their own practice; and track how their work may be contributing to changes in relationships and the way evidence is applied in local government settings. The markers of change and examples that we have identified can provide a useful reference point for embedded researchers, and in future evaluations of embedded researcher initiatives. Firstly, the framework can be used to develop clearer theories of change, which has been noted in previous research as important. ^12^ Secondly, based on our own experiences of benefiting personally from the ‘safe space’ in carrying out this reflective research, we suggest that the framework could also provide personal benefits to established embedded researchers. These benefits are particularly relevant in addressing the wider recognised challenges and isolation faced by embedded public health researchers in their roles, ^18^ developing a greater sense of purpose and confidence in their roles. Thirdly, the framework might be helpful to funders and host organisations in developing clearer job specifications and objectives for embedded researchers, which would have the added benefit of ensuring that there is greater clarity and clearer expectations about what it is possible to do and deliver when embedded researchers start in their roles. ^17^

Importantly, when described in the literature, markers of success for embedded research are often focused on the long-term, referring to culture-change, increasing capacity, evidence-led decision-making, and greater research infrastructure. ^11^ These are broad, complex, and long-term changes, which are difficult for individual embedded researchers to address on their own or track progress towards. To the best of our knowledge, only one other study has reported short-term markers of organisational culture change in local government settings, identifying three outcomes of importance: increased awareness, increased interest in research and increased involvement in research. ^17^ These outcomes resonate closely with our findings. For example, increased awareness and interest in research relates to our marker of ‘mindsets that seek research and evidence’ and involvement in research to our marker of ‘actively assessing and applying evidence in policy and practice’. ^17^ We suggest that our work extends the work of Edwards et al., ^17^ drawing together rigorous reflections to provide details about relational strategies as well as practical markers of short-term change, with tangible examples that have been informed by our practice.

### Strengths and limitations

This paper provides novel insights into how embedded public health researchers and their employers may monitor the effect of their role on the outcomes of building relationships and how research and other forms of evidence are applied in policy and practice. We believe that the heterogeneity in the structure or level of embeddedness of our roles is a strength, as despite this, we have been able to highlight common strategies and outcomes. There are, however, limitations. We aimed to identify markers of success and therefore have not fully reflected on barriers and enablers of change, how they interact, and how they are experienced across projects and roles in different local government contexts. Furthermore, the framework is developed only from our perspective as embedded researchers, neglecting those of wider stakeholders or host organisations. A future collaborative approach would be beneficial to understand and integrate the perspectives of those commissioning and working with embedded public health researchers.

## Conclusion

The novel embedded public health researcher framework developed includes four relational strategies used by embedded researchers and markers of subsequent change which can be used and tested in future research, evaluation, and practice. The framework could be used to develop clearer theories of change for future initiatives, help embedded researchers develop greater sense of purpose and confidence in their roles and/or clearer job specifications and objectives. Future research should explore the framework's utility from multiple stakeholder perspectives and examine barriers and enablers to implementation across different local government contexts.

## Ethics approval and consent to participate

Ethics approval to conduct this research was obtained from the University of Suffolk Research Ethics Committee (RETH(S)23/011). Researchers provided informed written consent prior to participating in the research. The research was carried out in line with the Helsinki Declaration.

## Consent for publication

Not applicable.

## Disclaimer

Content and views expressed in this paper are those of the authors based on their embedded research roles and do not necessarily represent the views of the host organisations or funding bodies.

## Data Availability

The datasets generated and/or analysed during the current study are not publicly available as it has not been possible to de-identify/fully anonymise the data. Any queries about accessing the data can be directed to Dr Rob Southall-Edwards r.southall-edwards@uos.ac.uk. Repository name: The role of embedded public health researchers in local government: Identifying markers of changes in relationships and how evidence is applied in policy and practice. https://doi.org/10.17605/OSF.IO/D2KHW. ^19^ This project contains the following extended data: Reflective memo template. (Template used by each researcher to explore the topics of relationships, capacity and culture change, including examples of things we were working on, what this had led to and examples of perceived ‘successes’ or issues we experienced). Semi-structured interview guide for peer-interviews. (Interview guide for semi-structured one-to-one peer-to-peer interviews). Data are available under the terms of the Creative Commons CC-By Attribution 4.0 International.

## References

[1] Local Government Association: *Public health in local government: Celebrating 10 years of transformation.* Lond Local Gov Assoc;2022.

[2] World Health Organization: What is a healthy city?. 2024 [cited 2024 Sep 29]. Reference Source

[3] Hock E Scope A Booth A : *Research capacity at a local government level (REC@ LL): mapping review and rapid systematic review.* Sheff ScHARR;2020.

[4] Cheetham M Wiseman A Khazaeli B : Embedded research: a promising way to create evidence-informed impact in public health?. *J. Public Health.* 2018;40(suppl_1):i64–i70. 10.1093/pubmed/fdx125 29538721

[5] Coates D Mickan S : Challenges and enablers of the embedded researcher model. *J. Health Organ. Manag.* 2020;34(7):743–764. 10.1108/JHOM-02-2020-0043 32924369

[6] Lomas J : Essay: Using ‘Linkage And Exchange’To Move Research Into Policy At A Canadian Foundation: Encouraging partnerships between researchers and policymakers is the goal of a promising new Canadian initiative. *Health Aff. (Millwood).* 2000;19(3):236–240. 10812803 10.1377/hlthaff.19.3.236

[7] Denis JL Lomas J : Convergent evolution: the academic and policy roots of collaborative research. *J. Health Serv. Res. Policy.* 2003;8 Suppl 2:1–6. 14596741 10.1258/135581903322405108

[8] Armstrong R Waters E Dobbins M : Knowledge translation strategies to improve the use of evidence in public health decision making in local government: intervention design and implementation plan. *Implement. Sci.* 2013;8:1–10. 24107358 10.1186/1748-5908-8-121 PMC3853093

[9] Barnes A Parkhurst J : Can Global Health Policy be Depoliticized? A Critique of Global Calls for Evidence‐Based Policy. *Handb. Glob. Health Policy.* 2014;157–173. 10.1002/9781118509623.ch8

[10] Orton L Lloyd-Williams F Taylor-Robinson D : The use of research evidence in public health decision making processes: systematic review. *PloS One.* 2011;6(7):e21704. 10.1371/journal.pone.0021704 21818262 PMC3144216

[11] Vindrola-Padros C Eyre L Baxter H : Addressing the challenges of knowledge co-production in quality improvement: learning from the implementation of the researcher-in-residence model. *BMJ Qual. Saf.* 2019;28(1):67–73. 10.1136/bmjqs-2017-007127 29866766 PMC6373423

[12] Kneale D Stansfield C Goldman R : The implementation of embedded researchers in policy, public services, and commercial settings: a systematic evidence and gap map. *Implement. Sci. Commun.* 2024;5(1):41. 10.1186/s43058-024-00570-3 38627834 PMC11020794

[13] Ward V Tooman T Reid B : Embedding researchers into organisations: a study of the features of embedded research initiatives. *Evid. Policy.* 2021;17(4):593–614.

[14] McGinity R Salokangas M : Introduction: ‘embedded research’ as an approach into academia for emerging researchers. *Manag. Educ.* 2014;28(1):3–5.

[15] Potts AJ Nobles J Shearn K : Embedded researchers as part of a whole systems approach to physical activity: reflections and recommendations. *Systems.* 2022;10(3):69.

[16] Newbury-Birch D Harbin K Adamson A : Establishing Research Ecosystems in Local Government: Ten lessons from the front line of the first year of the NIHR Health Determinants Research Collaborations (HDRCs). 2024 Mar [cited 2024 Oct 22]. Reference Source

[17] Edwards RC Kneale D Stansfield C : What are the mechanisms driving the early stages of embedded researcher interventions? A qualitative process evaluation in English local government. *Soc. Sci. Med.* 2024 Jan 1;340:116407. 10.1016/j.socscimed.2023.116407 38016307

[18] Woodall J Potts A Brown S : Embedded researchers in public health: a critical assessment. *Perspect. Public Health.* 2024;17579139231223711. 10.1177/17579139231223711 PMC13091917 38279198

[19] Dowling L : The role of embedded public health researchers in local government: Identifying markers of changes in relationships and how evidence is applied in policy and practice. 2026, February 3. 10.17605/OSF.IO/D2KHW PMC13324362 42403950

