# Supplementary material for: The role of embedded public health researchers in local government: Identifying markers of changes in relationships and how evidence is applied in policy and practice
Source: Wellcome Open Res. 2026 May 7;11:133. Originally published 2026 Feb 20. [Version 2] doi: 10.12688/wellcomeopenres.25838.2 (PMC13324362; doi:10.12688/wellcomeopenres.25838.2)
Supplement: Peer review history

# Reviewer report: Reviewer response for version 2

**DOI:** 10.21956/wellcomeopenres.29210.r158522

**Published:** 2026-07-03

**Authors:** Maria Catherine B Otero

**Affiliations:** 1 Department of Biological Sciences and Environmental Studies, University of the Philippines Mindanao College of Science and Mathematics (Ringgold ID: 482611), Davao City, Davao Region, Philippines; 2 Department of Clinical Epidemiology, College of Medicine, University of the Philippines Manila, Manila, Metro Manila, Philippines

Abstract: …This study aims to: (i) describe the rationale for our ER roles, (ii) understand strategies ERs use, and (iii) develop a framework to track changes resulting from ER roles.

 …Four key relational strategies by ERs were identified to enable change: (1) reflect on context and tailor communication approaches, (2) collaborate across boundaries, (3) be visible/accessible, and (4) create space to reflect and learn.

 Revised version of sentences in the abstract:   … evaluation, and practice to develop clearer theories of change for ER initiatives, help ERs develop confidence in their roles, and/or develop clearer ER job specifications and objectives.

 —— Findings: Figure 1 is redundant with table 1. Please remove table 1 and refer to figure 1 when discussing practical examples of markers of change.

 You mention the framework several times but I am not sure what you mean. Does this refer to the key relational strategies? Or is it figure 1? Please make a new figure highlighting this framework so readers can easily see it, or consider changing the caption of figure 1.

Is the work clearly and accurately presented and does it cite the current literature?

Yes

If applicable, is the statistical analysis and its interpretation appropriate?

Not applicable

Are all the source data underlying the results available to ensure full reproducibility?

No

Is the study design appropriate and is the work technically sound?

Yes

Are the conclusions drawn adequately supported by the results?

Yes

Are sufficient details of methods and analysis provided to allow replication by others?

Yes

Reviewer Expertise:

Public Health, Infectious Diseases Epidemiology, Medical Microbiology

I confirm that I have read this submission and believe that I have an appropriate level of expertise to confirm that it is of an acceptable scientific standard.

# Reviewer report: Reviewer response for version 2

**DOI:** 10.21956/wellcomeopenres.29210.r156368

**Published:** 2026-07-01

**Authors:** Maureen Dobbins

**Affiliations:** 1 McMaster University, Hamilton, Ontario, Canada

Description and evaluation of embedded researchers in public health is an important and timely topic as national research funders and jurisdictions around the world are contemplating initiating such arrangements. The findings of this study have potential to help inform these initiatives, and to identify strategies for developing and implementing such roles, and evaluating impact, particularly in the short to medium term. 

 The methods are appropriate and adequately described. The results are fulsome and clear. 

 The only substantial comments for improvement I have for this paper relate to the Discussion section. The authors state that very little is written about embedded researchers. However I disagree. A quick search of pubmed using embedded researcher as a text word yielded 49 papers, most if not all of which looked relevant. In addition, other roles, while slightly different, such as knowledge brokers, change champions, intermediaries, for which there is considerable literature, likely provide a good starting point, as these roles share many similarities in terms of roles and responsibilities, and indicators of success. 

 Particularly for knowledge brokers/brokering, there are some key systematic reviews that could be reviewed and cited. My sense is that more thinking could and should be done to compare and contrast the findings of this study with the relevant evidence base, which I suggest goes beyond the very narrow focus on considering research specific to the embedded research role.

Is the work clearly and accurately presented and does it cite the current literature?

Partly

If applicable, is the statistical analysis and its interpretation appropriate?

Not applicable

Are all the source data underlying the results available to ensure full reproducibility?

Yes

Is the study design appropriate and is the work technically sound?

Yes

Are the conclusions drawn adequately supported by the results?

Yes

Are sufficient details of methods and analysis provided to allow replication by others?

Yes

Reviewer Expertise:

Evidence-informed decision making, public health, knowledge brokers, knowledge translation

I confirm that I have read this submission and believe that I have an appropriate level of expertise to confirm that it is of an acceptable scientific standard, however I have significant reservations, as outlined above.

# Reviewer report: Reviewer response for version 1

**DOI:** 10.21956/wellcomeopenres.28439.r149052

**Published:** 2026-04-15

**Authors:** Anna Le Gouais

**Affiliations:** 1 University of Bristol, Bristol, UK

This is a well written paper which provides useful insights into embedded research. I support its indexing, and just provide a few relatively minor comments/suggestions below.

 Abstract:

 The abstract has the first study aim as: 'better understand the rationale for ERs’, or 'examine the purpose of ERs in local government’, however this doesn’t seem to align with the results, which identifies four relational strategies. Perhaps the aim needs to be re-phrased (e.g. about strategies?), as the intro already says they are needed to increase use of evidence. The introduction section talks about 'culture change, increasing capacity for evidence-informed decision-making, and greater research infrastructure’, which seems to be the assumed rationale for ERs in this paper. Other ERs may have different rationales (e.g. I’ve previously written about my experience of co-production of interventions (Le Gouais et al)).

 In the plain language abstract I suggest changing 'evidence-based’ to 'evidence-informed’, as there are other influencing factors beyond evidence in local government (this term is used later).

 Introduction:

 It’d be helpful to briefly outline what the 'Six different types of partnership structure’ mentioned are.

 Table 1 – Can you add who is funding these roles? How many are from HDRCs (mentioned in the intro)? Can you clarify if 'full time’ means only working in the local authority setting (5 days/week), or does it mean full time with some time in the local authority and some time at a university? Can this be clearer, especially if there is a split? I think this is useful information. I’m also interested that it says 'max one day per week on local government site’ for some – what % does this equate to? And how does that compare with 'normal’ local government officers? Finding 3. talks about being visible so this context seems important.

 I don’t recommend referencing Braun and Clarke 2006 and using the phrase 'codes that emerged’ or 'themes that emerged' – please see their more recent work on reflexive thematic analysis. This article also outlines the issue: https://journals.sagepub.com/doi/10.1177/14733250241277355 i.e. "...Braun and Clarke’s approach to thematic analysis, making clear that themes do not emerge from the data"

 Findings:

 The examples are clearly beyond traditional public health sectors (e.g. education, cycling). This could be made clearer earlier in the paper, e.g. Introduction.

 Figure 1 appears to be your theory of change – would it be helpful to describe it as this? In the discussion you say ' the framework can be used to develop clearer theories of change, which has been noted in previous research as important’ – do you mean your theory of change could be tested? If described as a theory of change (or logic model) then the long-term outcomes presumably would be 'culture-change, increasing capacity, evidence-led decision-making, and greater research infrastructure’. The framework in this paper therefore describes the short-term outcomes.

 You seem to repeat the table in Table 2 and I’m not clear why if it’s already in Figure 1.

 The markers of change, as described, appear based on perceptions. Do you think funders would be satisfied with these as 'evidence of impact’? Would you recommend ways to measure this if used in future (e.g. pre/post surveys)?

 The examples included for 'Actively accessing and applying evidence in policy and practice’ seem to describe methodological support to local government stuff, which is slightly different. Are there other examples you could draw on, or does the title need changing?

 'Limits to change’ – are you able to provide examples? Censorship sounds like a decision to hide evidence, whereas that’s quite different from lack of resources to enable change despite evidence.

Is the work clearly and accurately presented and does it cite the current literature?

Yes

If applicable, is the statistical analysis and its interpretation appropriate?

Not applicable

Are all the source data underlying the results available to ensure full reproducibility?

No source data required

Is the study design appropriate and is the work technically sound?

Yes

Are the conclusions drawn adequately supported by the results?

Yes

Are sufficient details of methods and analysis provided to allow replication by others?

Yes

Reviewer Expertise:

Embedded research, healthy urban development and sustainable transport (local government context)

I confirm that I have read this submission and believe that I have an appropriate level of expertise to confirm that it is of an acceptable scientific standard, however I have significant reservations, as outlined above.

# Response

**Published:** 2026-04-22

**Authors:** Lisa Dowling

**Affiliations:** Healthy Urban Places, Bradford Institute for Health Research, Bradford, England, UK

Dear Dr Le Gouais, Thank you for your positive comments on the manuscript and suggested minor amendments. We have been through each systemically and have indicated the changes that we have made in the text. We are pleased that we have been able to make these clarifications to improve the paper further.  

**COMMENT 1:** The abstract has the first study aim as: ‘better understand the rationale for ERs’, or ‘examine the purpose of ERs in local government’, however this doesn’t seem to align with the results, which identifies four relational strategies. Perhaps the aim needs to be re-phrased (e.g. about strategies?), as the intro already says they are needed to increase use of evidence. The introduction section talks about ‘culture change, increasing capacity for evidence-informed decision-making, and greater research infrastructure’, which seems to be the assumed rationale for ERs in this paper. Other ERs may have different rationales (e.g. I’ve previously written about my experience of co-production of interventions (Le Gouais et al)).        

**RESPONSE 1:** Thank you for highlighting the disconnect between the stated aims and the findings. We’ve clarified the aims in the paper to acknowledge that a key focus was on the strategies and framework and clarifying that ‘describing the rationale’ was one part of the whole study. We have also highlighted that our findings relating to our aim to describe the rationale for our roles is in the section “Introducing our ER roles – ways of working and rationale” These are the areas in which we have made changes to clarify: Abstract “Aims: to (i) describe the rationale for our ER roles, (ii) better understand strategies ERs use, (iii) put forward a framework to track changes resulting from ER roles” Plain language summary: “This study aimed to (1) examine the purpose of our ERs roles, (2) describe strategies ERs use, (3) develop a framework to track short-term changes resulting from ER work.” Introduction “We aim to (i) describe what we, as embedded researchers, identify as the common rationale for our work despite the heterogeneity of our roles, (ii) better understand the strategies ERs use to promote change and (iii) put forward a novel framework that we suggest can be used to track short-term changes resulting from embedded researcher roles, thus enabling a better understanding of progress towards longer-term outcomes of evidence-informed decision-making.”

**COMMENT 2: **In the plain language abstract I suggest changing ‘evidence-based’ to ‘evidence-informed’, as there are other influencing factors beyond evidence in local government (this term is used later).

**RESPONSE 2: **We have amended the language in the plain language summary and throughout the manuscript.

**COMMENT 3**: It’d be helpful to briefly outline what the ‘Six different types of partnership structure’ mentioned are.              

**RESPONSE 3:** Unfortunately, we do not have the space/word count to elaborate more fully on these partnership structures and also feel that doing so would draw focus from the aim of our paper. We have, however, given examples for the reader should they wish to review the reference in full.

**COMMENT 4:** Table 1 – Can you add who is funding these roles? How many are from HDRCs (mentioned in the intro)? Can you clarify if ‘full time’ means only working in the local authority setting (5 days/week), or does it mean full time with some time in the local authority and some time at a university? Can this be clearer, especially if there is a split? I think this is useful information. I’m also interested that it says ‘max one day per week on local government site’ for some – what % does this equate to? And how does that compare with ‘normal’ local government officers? Finding 3. talks about being visible so this context seems important.        

**RESPONSE 4: Thank** you for highlighting the need for clarification in our table. We understand hybrid may be confusing for readers in this context and have chosen to instead say remote / on-site as we are referring to our physical presence rather than a split between university and local authority. We have added funding to the table section “role type” and moved details which relate to our level of embeddedness (full or part time, remote / on-site) to the section “embedded role”. We have also added text to the findings as outlined below. “However, we worked in different types of local government settings (e.g. metropolitan district council vs. county council), had different funders (e.g. research, local authority, charity), had different levels of ‘embeddedness’ as a researcher.” “Three of us were Senior Research Fellows and two of us were Evaluation Researchers; we all worked our full contracted hours as embedded researchers.” We cannot comment on normal local government hours, different individuals / teams worked in different ways as although all were hybrid (remote and on-site), hours and days were different so it is difficult to estimate and compare.

**COMMENT 5: **I don’t recommend referencing Braun and Clarke 2006 and using the phrase ‘codes that emerged’ – please see their more recent work on reflexive thematic analysis. **RESPONSE 5: **We have used the updated reference as suggested and rephrased ‘codes that emerged’ to ‘themes’ (within the methods section).

**COMMENT 6: **The examples are clearly beyond traditional public health sectors (e.g. education, cycling). This could be made clearer earlier in the paper, e.g. Introduction. At the start of the paper, we have already included a sentence about the wider determinants of health including references which indicate that the wider determinants of health (to which our examples sometimes relate e.g. education, cycling) are a core part of public health. Figure 1 appears to be your theory of change – would it be helpful to describe it as this?  In the discussion you say ‘ the framework can be used to develop clearer theories of change, which has been noted in previous research as important’ – do you mean your theory of change could be tested? If described as a theory of change (or logic model) then the long-term outcomes presumably would be ‘culture-change, increasing capacity, evidence-led decision-making, and greater research infrastructure’. The framework in this paper therefore describes the short-term outcomes.

**RESPONSE 6: **We did consider throughout the study whether we aimed to create a theory of change or to identify changes. We felt that there was a gap in relation to understanding the short-term changes that ERs bring about and therefore focused on this. We believe that our results are better described as a framework as we are missing key components of a comprehensive theory of change (e.g. inputs, assumptions, details about the wider context, any feedback loops – though recognise that these do not always appear in a theory of change) and we did not set out to identify these. Our results may however be informative to others who want to create a theory of change for ERs.

**COMMENT 7:  **You seem to repeat the table in Table 2 and I’m not clear why if it’s already in Figure 1.   

**RESPONSE 7: **We acknowledge that there is repetition between Table 2 and Figure 1 however we wanted to provide different format for readers to ensure useability and accessibility of our findings.

**COMMENT 8: **The markers of change, as described, appear based on perceptions. Do you think funders would be satisfied with these as ‘evidence of impact’? Would you recommend ways to measure this if used in future (e.g. pre/post surveys)? Thank you – this is definitely an interesting point, but we feel it is beyond the scope of our study. We feel that significant consideration should be given to any measures to avoid any negative impacts on the relationships which ERs work so hard to establish.

**RESPONSE 8**: In our discussion we have added in a sentence to say: “This would enable the framework to be used in future evaluations, however, more work would be needed to identify limits to change in the context in which ERs are working and develop qualitative and quantitative measures for each of the markers, being mindful that whatever is used should not affect the relationships which ERs work so hard to cultivate..”

**COMMENT 9: **The examples included for ‘Actively accessing and applying evidence in policy and practice’ seem to describe methodological support to local government stuff, which is slightly different. Are there other examples you could draw on, or does the title need changing?

**RESPONSE 9: **We have clarified the role of evidence in our first example (see below) and feel that our second example is already a clear example of colleagues actively accessing (requesting a literature review) and applying evidence (informed a new local strategy), however, have clarified this further: “Here, one of us described how we had worked with a public health specialist to draw on evidence to improve the uptake of a survey about early years development that was lengthy and for which parental completion rates were low (Res3). Through drawing on evidence about question phrasing, recruitment processes, cultural appropriateness in asking probing questions and critical thinking together” Similarly, another of us shared an example of preparing a review that had been requested by the adult social care team to understand evidence about factors that shaped support for minoritised groups which actively informed a new local strategy (Res1).

**COMMENT 10: **‘Limits to change’ – are you able to provide examples?  Censorship sounds like a decision to hide evidence, whereas that’s quite different from lack of resources to enable change despite evidence.         

**RESPONSE 10: **We didn’t set out to explore limits to change within our study but, because we feel that this was an important point to raise, we have included a concise section in the manuscript. As it was not an aim of the study we only gave general examples due to our data collection methods and so as not to shift focus from our aims. We have highlighted that further research is needed in this area in our discussion:“This would enable the framework to be used in future evaluations however more work would be needed to identify limits to change …..”.   Kindest regards, Dr Lisa Dowling and Dr Amy Barnes on behalf of the research team.

# Reviewer report: Reviewer response for version 1

**DOI:** 10.21956/wellcomeopenres.28439.r149055

**Published:** 2026-03-17

**Authors:** Alexandra J Potts

**Affiliations:** 1 Leeds Beckett University, Leeds, UK

Thank you for allowing me the opportunity to review your manuscript. It is indeed a very important and timely topic and has the potential to influence working practices. I really enjoyed reading your work and could really resonate with the experiences shared. As you have highlighted in your strengths and limitations section, it would be good to explore the barriers or enablers of change (a part two paper perhaps?) as when I was reading your work this was the thing that stood out to me as being absent from your work - particularly given these are challenging aspects of the embedded researcher role. Aside from that point, I do not have any revisions or further suggestions for this paper. I think the authors have done an excellent job of sharing their experiences in a way which will sit well with both academics and individuals who are looking to have an embedded researcher as part of their work. Well done!

Is the work clearly and accurately presented and does it cite the current literature?

Yes

If applicable, is the statistical analysis and its interpretation appropriate?

Yes

Are all the source data underlying the results available to ensure full reproducibility?

Yes

Is the study design appropriate and is the work technically sound?

Yes

Are the conclusions drawn adequately supported by the results?

Yes

Are sufficient details of methods and analysis provided to allow replication by others?

Yes

Reviewer Expertise:

Public health, whole-systems approaches, embedded researcher

I confirm that I have read this submission and believe that I have an appropriate level of expertise to confirm that it is of an acceptable scientific standard.
